# Blood pressure status affects atrial fibrillation in diabetic end-stage renal disease

**DOI:** 10.1371/journal.pone.0283875

**Published:** 2023-04-04

**Authors:** Kyung-Do Han, YouMi Hwang, Sang Hyun Park

**Affiliations:** 1 Statistics and Actuarial Science, Soongsil University, Seoul, Republic of Korea; 2 Department of Cardiology, St. Vincent’s Hospital, The Catholic University of Korea, Seoul, Republic of Korea; 3 Catholic Research Institute for Intractable Cardiovascular Disease (CRID), College of Medicine, The Catholic University of Korea, Seoul, Republic of Korea; 4 Department of Medical Statistics, College of Medicine, Catholic University of Korea, Seoul, Republic of Korea; Kurume University School of Medicine, JAPAN

## Abstract

**Introduction:**

The prevalence of atrial fibrillation (AF) is increasing as the elderly population continues to increase. Chronic kidney disease, diabetes, and hypertension are known risk factors for AF. Since multimorbidity exists in chronic kidney disease, it is difficult to determine the impact of hypertension alone. Furthermore, little is known about the impact of hypertension on predicting AF in diabetic end-stage renal disease (ESRD). Here, we evaluated the effect of differential blood pressure control on AF prevalence among the diabetic ESRD population.

**Methods:**

From the Korean National Health Insurance Service database, 2 717 072 individuals with diabetes underwent health examinations during 2005–2019. Exactly 13 859 individuals with diabetic ESRD without a prior history of AF were selected and included in the analysis. Based on blood pressure level and previous hypertension medication history, we subdivided them into five groups: normal (normotensive), pre-hypertension, new onset hypertension, controlled hypertension, and uncontrolled hypertension. AF risk according to the blood pressure groups was estimated using Cox proportional-hazards models.

**Results:**

Among the five groups, the new onset hypertension, controlled hypertension, and uncontrolled hypertension groups showed a higher AF risk. In patients on antihypertensives, diastolic blood pressure ≥100 mmHg was significantly associated with AF risk. High pulse pressure showed a significant risk for AF in patients on antihypertensives.

**Conclusion:**

In patients with diabetic ESRD, overt hypertension and a history of hypertension impacts AF. AF risk was higher in the ESRD population with diastolic blood pressure ≥100 mmHg and pulse pressure >60 mmHg.

## Introduction

Atrial fibrillation (AF) is the most common arrhythmia diagnosed in adults. It tends to increase in prevalence as an aging population increases, and medical expenses simultaneously increase as a result [1]. Due to the serious complications of AF, its diagnosis and prevention are very important. Aging, hypertension, diabetes mellitus (DM), history of thromboembolism, heart failure, and vascular disease are all known risk factors for AF [2, 3]. These diseases often co-exist in the elderly. Chronic renal failure (CRF) is also one of the risk factors for AF, and its prevalence is also increasing [4, 5]. Among patients with CRF, it is known that the prevalence of AF increases in patients with end-stage renal disease (ESRD) [6, 7]. As the estimated glomerular filtration rate (eGFR) declines, there is upregulation of the renin-angiotensin-aldosterone system [8]. Water and salt retention occurs, which can lead to an increase in blood pressure (BP). Further, in advanced CRF, endothelial dysfunction and enhanced oxidative stress can be potential mechanisms for elevated BP and progression to ESRD [9, 10]. However, a consensus regarding the optimal BP target is not currently available for patients with CRF. Target BP goals can be changed as an individual ages or if underlying conditions exist, especially in patients with ESRD.

Although ESRD is a known risk factor for AF, we attempted to find the major factors involved in the development of AF in these patients. To compare the risk of AF by BP status, we decided to analyze diabetic patients with ESRD. This study aimed to elucidate the impact of BP on developing AF in diabetes patients with ESRD and to further provide some guidelines for BP management in patients with ESRD to lower AF development.

## Methods

### Data source

The Korean National Health Insurance Service database (from 2005 to 2019) was used for this study. The Korean National Health Insurance Service is a single national insurer and includes comprehensive information with overall medical coverage. This service has approximately 50 million individuals enrolled from the Republic of Korea, and data from this population include sociodemographic data, medical expenses, and diagnoses encoded by the *International Classification of Disease*, *Tenth Revision of Clinical Modification*. Korean National Health Insurance Service data also cover inpatient and outpatient clinic services, medical costs, pharmacy claims, and mortality information. Since the National Health Insurance Corporation recommends and provides a national general medical examination for the early detection of chronic diseases for adults over the age of 20 years, this examination includes physical examinations by a primary physician, blood tests, chest radiography, and self-reporting questionnaires regarding social and medical history. These results are contained in the National Health Insurance Service database and were provided anonymously. This study was conducted according to the Declaration of Helsinki and approved by the Institutional Review Board of St. Vincent’s Hospital, The Catholic University of Korea (Suwon, Republic of Korea; Institutional Review Board No. VC22ZASI0032). Regarding the retrospective nature of the data, informed consent was waived, which was also approved by the Institutional Review Board of St. Vincent’s Hospital, The Catholic University of Korea.

### Study population

**Fig 1:**
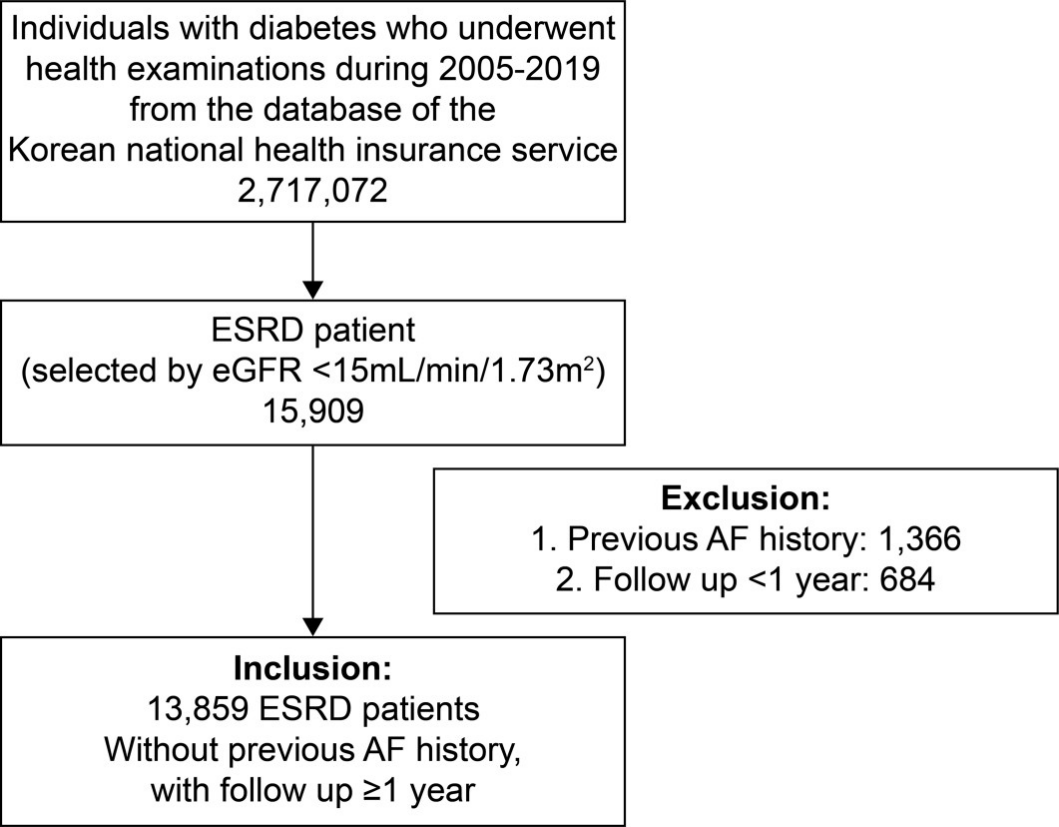
Participant selection flow chart.

Initially, a total of 2 717 072 individuals with diabetes who underwent health examinations from 2005 to 2019 were selected from the Korean National Health Insurance Service database. BP measurements were taken by a sphygmomanometer per published guidelines [11], and pulse pressure (PP) was calculated as the difference between systolic and diastolic blood pressure (SBP and DBP). AF was defined by a new clinical diagnosis recorded in claim data. A diagnosis in claim data required a single-entry code of I48.0 to I48.4, I48.91, or I48.92 in the *International Classification of Disease*, *Tenth Revision of Clinical Modification*. Body mass index (BMI) was calculated as a person’s weight in kilograms divided by the square of height in meters. In the relevant diabetic population (n = 2 717 072), we selected 15 909 patients with ESRD based on eGFR <15 mL/min/1.73 m^2^ and excluded 1366 patients with previous AF history and 684 who followed up less than 1 year later. Overall, 13 859 ESRD patients with diabetes were included in the analysis (Fig 1).

### Study design

Based on BP status and history of antihypertensive medication use, we classified the patients into five BP groups: normal group on no antihypertensive medication + SBP <120 mmHg and DBP <80 mmHg; pre-hypertension group on no antihypertensive medication + 120 mmHg ≤SBP <140 mmHg and 80 mmHg ≤DBP <90 mmHg; new onset hypertension group on no antihypertensive medication + SBP ≥140 mmHg or DBP ≥90 mmHg; controlled hypertension group on antihypertensive medication + SBP <140 mmHg and DBP <90 mmHg; and uncontrolled hypertension group on antihypertensive medication + SBP ≥140 mmHg or DBP ≥90 mmHg.

### Statistical analyses

Continuous variables are described as means ± standard deviations, and categorical variables are described as numbers and percentages. Across the five groups, baseline characteristics were analyzed. The incidence of newly developed AF was analyzed by dividing the number of events during the follow-up period by 1000 person-years at risk. The AF risk in relation to the hypertension levels was analyzed via survival analysis, using the log-rank test and Kaplan-Meier method. The hazard ratio (HR) and 95% confidence intervals (CIs) for AF were analyzed by Cox proportional-hazards regression. The non-adjusted analysis was presented as Model 1, and after adjustment for age and sex, it was presented as Model 2. After adjusting for age, sex, BMI, dyslipidemia, and smoking/alcohol consumption, it was presented as Model 3. Model 4 was further adjusted for age, sex, BMI, smoking, alcohol consumption, insulin treatment, number (≥3) of antidiabetic medications used, and duration (≥5 years) of diabetes condition. Subgroup analyses for SBP, DBP, and PP were conducted, and patients with or without antihypertensive medications were also analyzed. All the analyses were two-tailed, and *P* < 0.05 was considered significant. Statistical analyses were performed using SAS version 9.4 (SAS Institute Inc., Cary, NC, USA).

## Results

### Patient characteristics

The average ages of patients in the normal, pre-hypertension, new onset hypertension, controlled hypertension, and uncontrolled hypertension groups were 56.7 ± 11.4 (n = 880), 57.1 ± 11.2 (n = 1535), 58.4 ± 11.8 (n = 740), 60.7 ± 10.7 (n = 6383), and 61.4 ± 10.4 years (n = 4321), respectively. More than 50% of the study population was male. The percentages of individuals with diabetes conditions for more than 5 years in the normal, pre-hypertension, new onset hypertension, controlled hypertension, and uncontrolled hypertension groups were 51.7%, 49.0%, 52.7%, 65.0%, and 71.0%, respectively. Overall, 75% of the patients were on hemodialysis. Compared with the normal and pre-hypertensive groups, the other three groups showed higher BMI, higher waist circumferences, a high proportion of insulin treatment, and lower high-density lipoprotein levels. Baseline characteristics of the study population according to the five groups are shown in Table 1.

**Table 1:** Baseline characteristics in the five groups.

	1	2	3	4	5	*P* value
**n = 13 859**	**880**	**1535**	**740**	**6383**	**4321**	
**Age**	56.7 ± 11.4	57.1 ± 11.2	58.4 ± 11.8	60.7 ± 10.7	61.4 ± 10.4	< 0.0001
**Male (%)**	479 (54.4)	960 (62.5)	449 (60.7)	3991 (62.5)	2648 (61.3)	0.0002
**Duration of diabetes mellitus ≥ 5 years (%)**	455 (51.7)	752 (49.0)	390 (52.7)	4149 (65.0)	3066 (71.0)	< 0.0001
**Insulin treatment (%)**	257 (38.7)	378 (34.8)	232 (46.6)	2927 (52.9)	2180 (57.4)	< 0.0001
**Number of oral antidiabetic medications ≥3 (%)**	134 (15.2)	232 (15.1)	71 (9.6)	1081 (16.9)	765 (17.7)	< 0.0001
**Hypertension treatment (%)**	0 (0)	0 (0)	0 (0)	6383 (100)	4321 (100)	
**Hemodialysis (%)**	661 (75.1)	1146 (74.7)	564 (76.2)	4887 (75.6)	3319 (76.8)	0.4276
**Smoking habit (%)**						< 0.0001
Non	554 (63.0)	905 (59.0)	441 (59.6)	3830 (60.0)	2698 (62.4)	
Ex	169 (19.2)	314 (20.5)	148 (20.0)	1587 (24.9)	984 (22.8)	
Current	157 (17.8)	316 (20.6)	151 (20.4)	966 (15.1)	639 (14.8)	
**Drinking habit (%)**						< 0.0001
Non	674 (76.6)	1065 (69.4)	550 (74.3)	5070 (79.4)	3528 (81.7)	
Drinker	179 (20.3)	362 (23.6)	152 (20.5)	1123 (17.6)	666 (15.4)	
Heavy drinker	27 (3.1)	108 (7.0)	38 (5.1)	190 (3.0)	127 (2.9)	
**Weight (kg)**	60.9 ± 11.0	63.7 ± 10.8	63.7 ± 12.0	63.9 ± 11.0	64.4 ± 11.4	< 0.0001
**Height (cm)**	161.8 ± 8.8	163.0 ± 9.1	161.8 ± 9.4	162.1 ± 8.8	161.6 ± 8.9	< 0.0001
**BMI (kg/m** ^**2**^ **)**	23.2 ± 3.3	23.9 ± 3.2	24.3 ± 3.6	24.3 ± 3.5	24.6 ± 3.5	< 0.0001
**Waist circumference (cm)**	81.3 ± 9.0	83.4 ± 8.4	84.1 ± 9.3	85.0 ± 9.2	86.0 ± 9.3	< 0.0001
**Systolic blood pressure (mmHg)**	108.6 ± 8.1	127.3 ± 6.9	150.2 ± 14.6	123.1 ± 10.9	152.3 ± 14.8	< 0.0001
**Diastolic blood pressure (mmHg)**	67.6 ± 6.6	77.5 ± 6.4	88.5 ± 10.1	73.7 ± 8.3	87.0 ± 11.0	< 0.0001
**Pulse pressure (mmHg)**	41.0 ± 6.5	49.8 ± 8.5	61.7 ± 14.9	49.4 ± 9.4	65.3 ± 15.3	< 0.0001
**Dyslipidemia**	375 (42.6)	662 (43.1)	306 (41.4)	3718 (58.3)	2505 (58.0)	< 0.0001
**Total cholesterol (mg/dl)**	188.5 ± 45.1	191.2 ± 46.0	195.7 ± 53.0	180.1 ± 44.6	185.7 ± 49.8	< 0.0001
**HDL (mg/dl)**	51.4 ± 14.7	51.5 ± 19.6	50.6 ± 15.7	48.8 ± 25.4	48.0 ± 22.2	< 0.0001
**LDL (mg/dl)**	109.0 ± 48.2	108.9 ± 42.5	112.2 ± 45.4	101.3 ± 42.6	106.2 ± 42.1	< 0.0001
TG*(mg/dl)	125 (87–177)	133 (94–194)	137 (95–197.5)	136 (96–193)	135 (97–198)	0.0342

**Abbreviations**: 1, normal group; 2, pre-hypertension group; 3, new onset hypertension group; 4, controlled hypertension group; 5, uncontrolled hypertension group; BMI, body mass index; HDL, high-density lipoprotein; LDL, low-density lipoprotein; TG, triglyceride.

*In the case of TG, it was expressed as the geometric mean (95% Cl), unlike other continuous variables, to show representativeness due to bias in the distribution.

### Clinical outcomes

**Fig 2:**
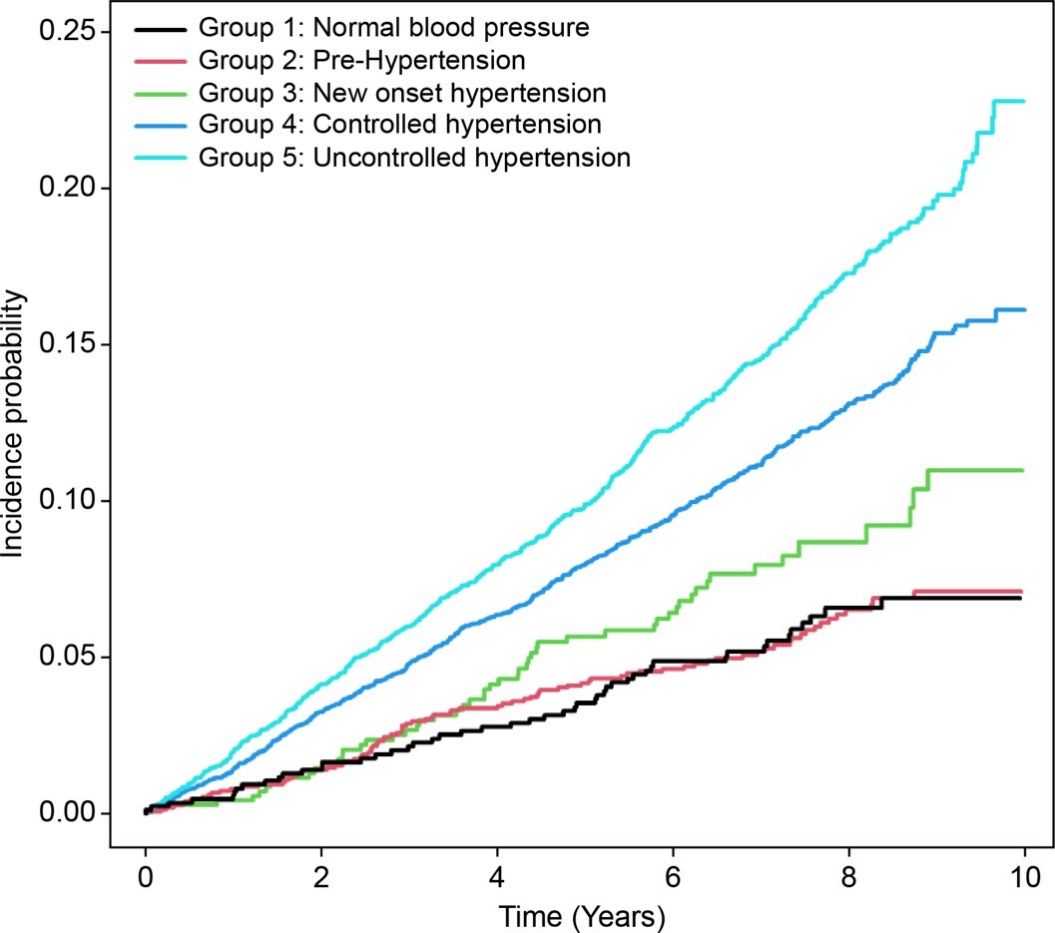
Atrial fibrillation risk according to blood pressure status (Model 4).

The trend of AF risk in each group was: Normal group < Pre-hypertension group < New onset hypertension group < Controlled hypertension group < Uncontrolled hypertension group (Table 2). In Models 1, 2, and 3, the HR of AF risk was significantly high in the hypertension treatment groups (Model 1: controlled hypertension HR = 2.264 [1.695–3.025], uncontrolled hypertension HR = 3.03 [2.264–4.055]; Model 2: controlled hypertension HR = 1.979 [1.48–2.645], uncontrolled hypertension HR = 2.62 [1.957–3.509]; Model 3: controlled hypertension HR = 1.949 [1.456–2.607], uncontrolled hypertension HR = 2.564 [1.912–3.438]). This result was also seen in Model 4 (controlled hypertension HR = 1.792 [1.295–2.482], uncontrolled hypertension HR = 2.291 [1.651–3.18]). In the final model, the new onset hypertension group had a statistically significant, substantially higher risk of AF than the normal and pre-hypertension groups (new onset hypertension HR = 1.609 [1.045–2.477]) (Fig 2).

**Table 2:** Atrial fibrillation risk according to the five groups.

	N	AF	Duration	IR(per 1000)	HR model1	HR model2	HR model3	HR model4
**1. Normal**	880	49	6332.81	7.7375	1(Ref.)	1(Ref.)	1(Ref.)	1(Ref.)
**2. Pre-hypertension**	1535	86	10910.67	7.8822	1.021(0.719,1.45)	0.984(0.692,1.397)	1.002(0.705,1.424)	1.013(0.681,1.507)
**3. New onset hypertension**	740	53	4720.41	11.2278	1.466(0.994,2.162)	1.39(0.943,2.05)	1.409(0.955,2.079)	1.609(1.045,2.477)
**4. Controlled hypertension**	6383	703	40487.84	17.3632	2.264(1.695,3.025)	1.979(1.48,2.645)	1.949(1.456,2.607)	1.792(1.295,2.482)
**5. Uncontrolled hypertension**	4321	596	25768.95	23.1286	3.03(2.264,4.055)	2.62(1.957,3.509)	2.564(1.912,3.438)	2.291(1.651,3.18)

**Abbreviations:** HR, hazard ratio.

*Model 1—Non-adjusted.

**Model 2—Adjusted for age and sex.

***Model 3—Adjusted for age, sex, BMI, smoking/alcohol consumption, and dyslipidemia.

****Model 4—Adjusted for age, sex, BMI, smoking/alcohol consumption, dyslipidemia, insulin treatment, number of antidiabetic medications used, and duration of diabetes condition.

A multivariate analysis according to the constituent factors of BP was performed. After adjusting for age, sex, BMI, smoking, drinking, insulin treatment, higher number (≥ 3) of antidiabetic medications used, and duration of diabetes (≥ 5 years), higher SBP showed a trend of increased AF risk without statistical significance. DBP > 100 mmHg (HR 1.26 [1.054–1.505]), PP between 60 and 80 mmHg (HR 1.359 [1.024–1.805]), and PP ≥ 80 mmHg (HR 1.601 [1.211–2.117]) were associated with an increased risk of AF (S1 Table).

In addition, AF risk was evaluated according to antihypertensive medication use. In patients on antihypertensive medications, DBP ≥ 100 mmHg was significantly associated with AF risk (HR 2.072 [1.195–3.591]). In patients on antihypertensive medication, high PP was significantly associated with AF risk (in PP between 60 and 80 mmHg, HR 1.40 [1.019–1.924] and PP ≥ 80 mmHg, HR 1.627 [1.19–2.223]) (S2 Table).

## Discussion

An increase in BP can accelerate a decrease in renal function, and the retention of water and salt also causes a further increase in BP. Further, endothelial dysfunction and arterial stiffness are common characteristics of diabetes and ESRD, which can cause diverse cardiovascular diseases, including AF [12]. These mechanisms can be factors that cause an increase in BP or a deterioration of renal function. Chronic elevation of BP can also cause end-organ damage. For instance, structural, hemodynamic, and electrophysiological alterations, such as ventricular hypertrophy can occur in the heart, leading to an increase in atrial size and diastolic dysfunction, creating conditions for AF development [13].

### Systolic pressure

In the ACCORD study, an intensive BP control group lowered the incidence of AF. In that study, the intensive BP control target was SBP < 120 mmHg [14]. However, treatment targeting systolic BP often complicates drug-induced and orthostatic hypotension and decreases both cardio-cerebral and renal perfusion, which can deteriorate renal function in patients with CRF. Cardiovascular mortality is higher in low SBP than normotensives in ESRD [15–19]. In our data, the effect of lower SBP (≤100 mmHg) was unclear since a small number of subjects were analyzed; however, we noticed that higher SBP was related to higher AF risk. Therefore, we can conclude that SBP < 140 mmHg significantly reduces the risk of AF development. Nonetheless, the benefits of target systolic BP < 120 mmHg in this population for cardiovascular mortality or stroke require more supporting data.

### Diastolic pressure

Although current guidelines for hypertension management suggest upper limits for both SBP and DBP, DBP is usually overlooked in clinics. It is reported that even in controlled hypertension, lower than normal or higher than normal DBP is associated with higher cardiovascular mortality and stroke [20, 21]. In general, DBP and SBP elevations are associated with aging. There can be isolated DBP elevation in the course of developing hypertension or diastolic hypertension alone, and even with isolated diastolic hypertension in a study, a higher cardiovascular outcome was reported [22]. This suggests that both SBP and DBP targets should be considered in real-world practice. In this study and analysis of the overall population, DBP >100 mmHg was associated with a higher risk for AF (S1 Table). In the subgroup analysis, subjects with a DBP of 90–99 mmHg without previous hypertension treatment showed a higher risk of AF development. In the subgroup, there was a significantly higher risk of AF for DBP ≥ 100 mmHg (S2 Table).

### Pulse pressure

PP, by definition, is affected by SBP and DBP. It is well known that as individuals age, PP increases due to arterial stiffening. Similar to SBP, PP exhibits a J-curve showing poor prognosis at lower and higher PP. Low PP particularly is a poor prognostic factor in both the elderly and patients with heart failure [23]. High PP is known to be associated with worsening of atherosclerosis [24, 25], and higher PP is related to higher SBP [26–28]. In our data, PP > 60 mmHg was related to a substantial risk for AF, especially in patients using antihypertensive medications. This suggests that PP is also an important prognostic factor during hypertension management in patients with diabetic ESRD.

### Optimized BP management in patients with ESRD and the importance of upstream treatment

Currently, with the development of novel oral anticoagulants (NOACs) and tremendous progress in ablation techniques, it has become possible to systematically manage and treat AF more effectively than in previous years. Optimizing treatment for the underlying disease must be prioritized as much as anticoagulant treatment for already diagnosed AF and rhythm control to restore or to maintain sinus rhythm. This is called upstream therapy. For example, optimized glycemic control and managing diabetic complications are required in patients with DM, and optimized BP management according to guidelines is also required in patients with high BP. Since various conditions may affect temporal BP, such as during hemodialysis or autonomic dysfunction with fluctuating BP according to medical treatment, there is a lack of consensus regarding the optimal BP target in CRF, especially in ESRD. In a study by Li et al. [17], higher pre-dialysis BP appeared to be protective, with the lowest mortality in SBP of 160–180 mmHg and pre-dialysis SBP < 120 mmHg showing the highest mortality in patients with ESRD.

Nonetheless, in our study, the new onset hypertension, controlled hypertension, and uncontrolled hypertension groups showed higher AF risk than the normotensive and pre-hypertension groups. This is interesting because even in patients with controlled hypertension (those on antihypertensive medication), the risk of AF was higher than in patients with normotension or pre-hypertension. The high risk of AF despite the BP being controlled with treatment suggests that prevention at an early stage of hypertension is more important. This, therefore, shows the importance of upstream treatment.

AF risk was especially higher in treated patients with PP > 60 mmHg. In patients without treatment, diastolic hypertension (≥ 100 mmHg) showed a higher AF risk. Based on the results of this study, the optimal BP target in ESRD to lower AF risk is suggested as 120 mmHg ≤ SBP < 140 mmHg and 80 mmHg ≤ DBP < 90 mmHg with PP < 60 mmHg (especially in patients undergoing treatment).

The Korean National Health Insurance Service is a unique, well-controlled and defined system. Individuals included in this system are recommended to undergo a general medical examination annually or biennially. Based on that dataset, we defined the BP status and medical history of enrolled patients and divided them into five BP levels. Nonetheless, this study has some limitations. First, although this was a large observational study, the setting was significantly different from that of a randomized controlled trial. Despite adjusting for various covariates, there may have been overlooked or unmeasurable confounders. Second, there was an inevitable limitation to the ascertainment of AF using the diagnostic codes rather than the electrocardiogram of each subject due to the nature of the data, although the definition of new-onset AF in the nationwide claims database had been validated and was generally used. Third, the measurement of BP during the health checkup might have been inaccurate due to a lack of repeat measurements and white coat hypertension. It could have been difficult to accurately determine a clinical problem from a faulty blood pressure measurement. Finally, the treatment differences in hypertension management may be meaningful in understanding the relationship between BP levels and outcomes. Although higher daytime BP or a history of hypertension are risk factors for AF, by the nature of these observational studies, treatment for hypertension was not randomly assigned or strictly controlled for the types of antihypertensive medications used or the duration of treatment. Therefore, in this study, attention should have been paid to the interpretation of the treatment effect in hypertensive patients. Future studies evaluating the effect of diurnal variation of BP on AF development and AF risk according to the duration of treatment or types of antihypertensive medications used in patients with ESRD are also needed.

In conclusion, untreated hypertension and a history of hypertension in patients with diabetic ESRD increase the risk of AF development. Controlled hypertension also appears to increase AF risk. AF risk was higher in the ESRD population with DBP > 100 mmHg and PP > 60 mmHg. Moreover, in patients using antihypertensive medications, PP ≥ 60 mmHg was a risk factor for AF. As DBP, SBP, and PP affect the occurrence of AF in patients with ESRD, DBP, SBP, and PP should also be emphasized respectively in the treatment target when monitoring and treating hypertension.

## Supporting information

S1 Table Atrial fibrillation risk according to constituent factors of blood pressure. (DOCX)

S2 Table Atrial fibrillation risk according to antihypertensive medication use. (DOCX)
