# Supplementary material for: Blood pressure status affects atrial fibrillation in diabetic end-stage renal disease
Source: PLoS One. 2023 Apr 4;18(4):e0283875. doi: 10.1371/journal.pone.0283875 (PMC10072463; doi:10.1371/journal.pone.0283875)
Supplement: S1 Table — (DOCX) [file pone.0283875.s001.docx]

**Supplementary Materials**

**Supplementary Table S1. Atrial fibrillation risk according to constituent factors of blood pressure**

|  | **n** | **AF** | **Model 1*** | **Model 2**** | **Model 3***** | **Model 4****** |
| --- | --- | --- | --- | --- | --- | --- |
| **SBP (mmHg)** |  |  |  |  |  |  |
| ≤100 | 244 | 23 | 1 (Ref.) | 1 (Ref.) | 1 (Ref.) | 1 (Ref.) |
| 101-119 | 2567 | 233 | 0.937 (0.61, 1.438) | 0.937 (0.611, 1.439) | 0.966 (0.629, 1.484) | 1.076 (0.671, 1.724) |
| 120-139 | 6361 | 608 | 1 (0.659, 1.516) | 0.954 (0.629, 1.446) | 0.945 (0.623, 1.435) | 1.129 (0.714, 1.786) |
| 140-159 | 3233 | 419 | 1.474 (0.969, 2.243) | 1.352 (0.888, 2.057) | 1.255 (0.823, 1.912) | 1.431 (0.901, 2.272) |
| ≥160 | 1454 | 204 | **1.666 (1.083, 2.564)** | **1.583 (1.028, 2.437)** | 1.448 (0.94, 2.232) | 1.568 (0.975, 2.519) |
| **DBP (mmHg)** |  |  |  |  |  |  |
| <70 | 2195 | 245 | 1 (Ref.) | 1 (Ref.) | 1(Ref.) | 1 (Ref.) |
| 70-89 | 4242 | 421 | **0.838 (0.716, 0.981)** | 0.896 (0.765, 1.049) | 0.906 (0.774, 1.062) | 0.985 (0.833, 1.164) |
| 90-99 | 4779 | 500 | 0.884 (0.759, 1.03) | 0.96 (0.823, 1.119) | 0.952 (0.816, 1.111) | 1.098 (0.933, 1.292) |
| ≥100 | 2643 | 321 | 1.071 (0.907, 1.265) | **1.184 (1.002, 1.399)** | 1.129 (0.954, 1.336) | **1.26 (1.054, 1.505)** |
| **PP (SBP-DBP) (mmHg)** |  |  |  |  |  |  |
| <40 | 977 | 66 | 1 (Ref.) | 1 (Ref.) | 1 (Ref.) | 1 (Ref.) |
| 40-60 | 3600 | 311 | 1.287 (0.987, 1.678) | 1.204 (0.923, 1.571) | 1.21 (0.927, 1.579) | 1.238 (0.926, 1.656) |
| 60-80 | 4317 | 444 | **1.603 (1.238, 2.076)** | **1.401 (1.081, 1.816)** | **1.335 (1.029, 1.731)** | **1.359 (1.024, 1.805)** |
| ≥ 80 | 4965 | 666 | **2.302 (1.787, 2.965)** | **1.897(1.471, 2.447)** | **1.716 (1.329, 2.215)** | **1.601 (1.211, 2.117)** |

Abbreviations: AF, atrial fibrillation; DBP, diastolic blood pressure; HR, hazard ratio; SBP, systolic blood pressure

*Model 1 – Non-adjusted

**Model 2 – Adjusted for age and sex

***Model 3 – Adjusted for age, sex, BMI, smoking/alcohol consumption, and dyslipidemia

****Model 4 – Adjusted for age, sex, BMI, smoking/alcohol consumption, dyslipidemia, insulin treatment, number of antidiabetic medications used, and duration of diabetes condition
