# Supplementary material for: Blood pressure status affects atrial fibrillation in diabetic end-stage renal disease
Source: PLoS One. 2023 Apr 4;18(4):e0283875. doi: 10.1371/journal.pone.0283875 (PMC10072463; doi:10.1371/journal.pone.0283875)
Supplement: S2 Table — (DOCX) [file pone.0283875.s002.docx]

**Supplementary Materials**

**Supplementary Table S2. Atrial fibrillation risk according to antihypertensive medication use**

| **No antihypertensive medication group** | | | | | |  |  |  |
| --- | --- | --- | --- | --- | --- | --- | --- | --- |
|  | **N** | **AF** | **Model 1*** | **Model 2**** | **Model 3***** | **Model 4****** |  |  |
| **SBP** |  |  |  |  |  |  |  |  |
| ≤100 | 85 | 7 | 1 (Ref.) | 1 (Ref.) | 1 (Ref.) | 1 (Ref.) |  |  |
| 101-119 | 856 | 42 | 0.55 (0.247, 1.224) | 0.558 (0.25, 1.245) | 0.584 (0.261, 1.308) | 0.921 (0.325, 2.606) |  |  |
| 120-139 | 1558 | 88 | 0.649 (0.301, 1.402) | 0.635 (0.293, 1.377) | 0.685 (0.314, 1.497) | 1.027 (0.371, 2.844) |  |  |
| 140-159 | 478 | 31 | 0.843 (0.371, 1.914) | 0.793 (0.348, 1.807) | 0.858 (0.374, 1.97) | 1.297 (0.449, 3.749) |  |  |
| ≥160 | 178 | 20 | 1.526 (0.645, 3.61) | 1.433 (0.604, 3.401) | 1.544 (0.646, 3.691) | 2.507 (0.824, 7.626) |  |  |
| **DBP** |  |  |  |  |  |  |  |  |
| <70 | 532 | 34 | 1 (Ref.) | 1 (Ref.) | 1 (Ref.) | 1 (Ref.) |  |  |
| 70-89 | 1096 | 58 | 0.788 (0.516, 1.204) | 0.84 (0.55, 1.284) | 0.87 (0.568, 1.332) | 1.022 (0.628, 1.663) |  |  |
| 90-99 | 1098 | 65 | 0.913 (0.603, 1.383) | 1.017 (0.67, 1.544) | 1.064 (0.699, 1.62) | 1.286 (0.796, 2.078) |  |  |
| ≥100 | 429 | 31 | 1.157 (0.711, 1.882) | 1.302 (0.799, 2.122) | 1.389 (0.848, 2.276) | **2.072 (1.195, 3.591)** |  |  |
| **PP(SBP-DBP)** |  |  |  |  |  |  |  |  |
| <40 | 335 | 16 | 1 (Ref.) | 1 (Ref.) | 1 (Ref.) | 1 (Ref.) |  |  |
| 40-60 | 1124 | 55 | 1.012 (0.58, 1.765) | 0.956 (0.547, 1.67) | 0.969 (0.554, 1.695) | 1.068 (0.562, 2.029) |  |  |
| 60-80 | 982 | 63 | 1.396 (0.807, 2.417) | 1.249 (0.72, 2.167) | 1.272 (0.731, 2.213) | 1.185 (0.621, 2.261) |  |  |
| ≥80 | 714 | 54 | 1.872 (1.071, 3.271) | 1.515 (0.861, 2.664) | 1.558 (0.884, 2.748) | 1.526 (0.796, 2.926) |  |  |
| **Antihypertensive medication group** | | | | | | |  |  |
|  | **N** | **AF** | **Model 1*** | **Model 2**** | **Model 3***** | **Model 4****** |  |  |
| **SBP** |  |  |  |  |  |  |  |  |
| ≤100 | 159 | 16 | 1 (Ref.) | 1 (Ref.) | 1 (Ref.) | 1 (Ref.) |  |  |
| 101-119 | 1711 | 191 | 1.111 (0.667, 1.85) | 1.13 (0.678, 1.882) | 1.154 (0.692, 1.922) | 1.12 (0.66, 1.903) |  |  |
| 120-139 | 4803 | 520 | 1.066 (0.648, 1.753) | 1.054 (0.641, 1.733) | 1.086 (0.66, 1.787) | 1.156 (0.691, 1.935) |  |  |
| 140-159 | 2755 | 388 | 1.477 (0.896, 2.436) | 1.423 (0.863, 2.346) | 1.445 (0.876, 2.384) | 1.453 (0.866, 2.438) |  |  |
| ≥160 | 1276 | 184 | 1.576 (0.946, 2.627) | 1.581 (0.949, 2.635) | 1.604 (0.962, 2.676) | 1.531 (0.902, 2.6) |  |  |
| **DBP** |  |  |  |  |  |  |  |  |
| <70 | 1663 | 211 | 1 (Ref.) | 1 (Ref.) | 1 (Ref.) | 1 (Ref.) |  |  |
| 70-89 | 3146 | 363 | 0.857 (0.723, 1.015) | 0.915 (0.772, 1.085) | 0.919 (0.775, 1.09) | 0.987 (0.826, 1.181) |  |  |
| 90-99 | 3681 | 435 | 0.863 (0.732, 1.017) | 0.936 (0.793, 1.104) | 0.943 (0.799, 1.113) | 1.075 (0.905, 1.279) |  |  |
| ≥100 | 2214 | 290 | 0.991 (0.83, 1.184) | 1.105 (0.924, 1.32) | 1.11 (0.928, 1.328) | 1.195 (0.99, 1.443) |  |  |
| **PP (SBP-DBP)** |  |  |  |  |  |  |  |  |
| <40 | 642 | 50 | 1 (Ref.) | 1 (Ref.) | 1 (Ref.) | 1 (Ref.) |  |  |
| 40-60 | 2476 | 256 | 1.339 (0.989, 1.814) | 1.266 (0.935, 1.714) | 1.287 (0.951, 1.744) | 1.283 (0.925, 1.78) |  |  |
| 60-80 | 3335 | 381 | 1.524 (1.135, 2.047) | 1.36 (1.012, 1.828) | 1.373 (1.021, 1.845) | **1.4 (1.019, 1.924)** |  |  |
| ≥80 | 4251 | 612 | 2.078(1.558,2.772) | 1.778 (1.331, 2.375) | 1.776 (1.329, 2.373) | **1.627 (1.19, 2.223)** |  |  |

Abbreviations: AF, atrial fibrillation; DBP, diastolic blood pressure; HR, hazard ratio; SBP, systolic blood pressure

*Model 1 – Non-adjusted

**Model 2 – Adjusted for age and sex

***Model 3 – Adjusted for age, sex, BMI, smoking/alcohol consumption, and dyslipidemia

****Model 4 – Adjusted for age, sex, BMI, smoking/alcohol consumption, dyslipidemia, insulin treatment, number of antidiabetic medications used, and duration of diabetes condition
